# Next-generation RNA sequencing of FFPE subsections reveals highly conserved stromal reprogramming between canine and human mammary carcinoma

**DOI:** 10.1242/dmm.040444

**Published:** 2019-08-08

**Authors:** Parisa Amini, Sina Nassiri, Julia Ettlin, Alexandra Malbon, Enni Markkanen

**Affiliations:** 1 Institute of Veterinary Pharmacology and Toxicology, Vetsuisse Faculty, University of Zürich, CH-8057 Zürich, Switzerland; 2 Bioinformatics Core Facility, Swiss Institute of Bioinformatics, CH-1015 Lausanne, Switzerland; 3 Institute of Veterinary Pathology, Vetsuisse Faculty, University of Zürich, CH-8057 Zürich, Switzerland

**Keywords:** Formalin-fixed paraffin embedded, RNAseq, Laser-capture microdissection, Canine mammary carcinoma, Breast cancer, Tumour stroma

## Abstract

Spontaneous canine simple mammary carcinomas (mCA) are often viewed as models of human mCA. Cancer-associated stroma (CAS) is central for initiation and progression of human cancer, and is likely to play a key role in canine tumours as well. However, canine CAS lacks characterisation and it remains unclear how canine and human CAS compare. Formalin-fixed paraffin embedded (FFPE) tissue constitutes a valuable resource of patient material, but chemical crosslinking has largely precluded its analysis by next-generation RNA sequencing (RNAseq). We have recently established a protocol to isolate CAS and normal stroma from archival FFPE tumours using laser-capture microdissection followed by RNAseq. Using this approach, we have analysed stroma from 15 canine mCA. Our data reveal strong reprogramming of canine CAS. We demonstrate a high-grade molecular homology between canine and human CAS, and show that enrichment of upregulated canine CAS genes strongly correlates with the enrichment of an independently derived human stromal signature in the TCGA breast tumour dataset. Relationships between different gene signatures observed in human breast cancer are largely maintained in the canine model, suggesting a close interspecies similarity in the network of cancer signalling circuitries. Finally, we establish the prognostic potential of the canine CAS signature in human samples, emphasising the relevance of studying canine CAS as a model of the human disease. In conclusion, we provide a proof-of-principle to analyse specific subsections of FFPE tissue by RNAseq, and compare stromal gene expression between human and canine mCA to reveal molecular drivers in CAS supporting tumour growth and malignancy.

## Abstract (precis)

**Summary:** This study offers proof-of-principle for a novel protocol to analyse gene expression in subsections of FFPE patient tissue, supporting the use of spontaneous canine mammary tumours as models for the human disease.

## INTRODUCTION

The microenvironment surrounding cancer cells is pivotal for growth and survival of many different tumours (Hanahan and Coussens, 2012). This so-called cancer-associated stroma (CAS), consisting of a mixture of different non-tumour cells such as immune cells, fibroblasts and others, as well as extracellular matrix, plays a key role in cancer initiation and progression (Hanahan and Coussens, 2012). The role of CAS in tumour biology has been widely documented (Bissell and Hines, 2011). CAS directly promotes the growth of tumour cells by secreting and/or activating molecules such as growth factors, nutrients and cytokines, among others (e.g. reviewed by Bissell and Hines, 2011; Hanahan and Coussens, 2012). Still, the mechanisms underlying the formation of CAS and the molecular dialogue between CAS and cancer cells remain poorly understood.

Based on the closely related pathophysiology, spontaneously occurring cancer in the domestic dog is increasingly viewed as a valuable model to foster understanding of cancer biology and potentially identify novel therapeutic targets in both dogs and human patients (Gardner et al., 2015; Karlsson and Lindblad-Toh, 2008; Rogers, 2015). In particular, owing to strong molecular and clinical similarities, canine mammary carcinoma (mCA) are regarded as excellent models for human mCA and are thought to overcome several of the limitations of xenograft or genetically modified rodent tumour models (Liu et al., 2014; Queiroga et al., 2011b; Schiffman and Breen, 2015). Histologically and at the molecular level, canine simple mCA closely replicate the biology of human mCA and mirror many of the genomic aberrations found therein, and are thus thought to reflect human mCA (Liu et al., 2014; Queiroga et al., 2011b). Also, as the most frequently occurring cancer in intact female dogs, canine mCA are highly relevant in the veterinary clinical setting (Salas et al., 2015). Canine simple mCA are classified as malignant epithelial neoplasms that infiltrate the surrounding mammary tissue, thus inducing a strong stromal response and myofibroblast proliferation, and can give rise to metastases (Goldschmidt et al., 2011).

Given the central role of CAS in the biology of human cancer in general, and mCA in particular, it is likely to also play a central role in the growth and development of canine mCA. Until now, however, despite the fact that canine cancer is increasingly analysed, canine CAS almost entirely lacks characterisation. Thus, it remains unclear whether CAS has a similar role in canine cancers, which mechanisms are involved in its formation and whether CAS from dogs is comparable to human CAS. Understanding the role of CAS in canine cancers and its comparability to human CAS is pivotal to define the validity of canine cancers as models for the human disease. Furthermore, a comparison of human and canine CAS can not only reveal key changes that are of central importance for the development of the disease itself, but also lead to identification of novel targets for pharmacological intervention against cancer.

Formalin-fixed paraffin embedded (FFPE) tissue constitutes a vast resource of patient material. However, FFPE heavily impacts on RNA quantity and quality, which proves challenging in downstream applications. To enable the use of this valuable tissue resource and analysis of CAS reprogramming in tumours, we have established isolation of CAS and matched normal stroma from canine simple mCA by laser-capture microdissection (LCM) from archival FFPE samples followed by analysis by next-generation RNA sequencing (RNAseq). Here, we provide the proof-of-principle for this novel protocol by analysing CAS and matched normal stroma isolated by LCM from FFPE tissue from 15 clinical cases of canine simple mCA.

## RESULTS

### RNAseq-based transcriptomic profiling of matched cancer-associated and normal stroma from canine mCA isolated by LCM from FFPE specimens

**Fig. 1.**
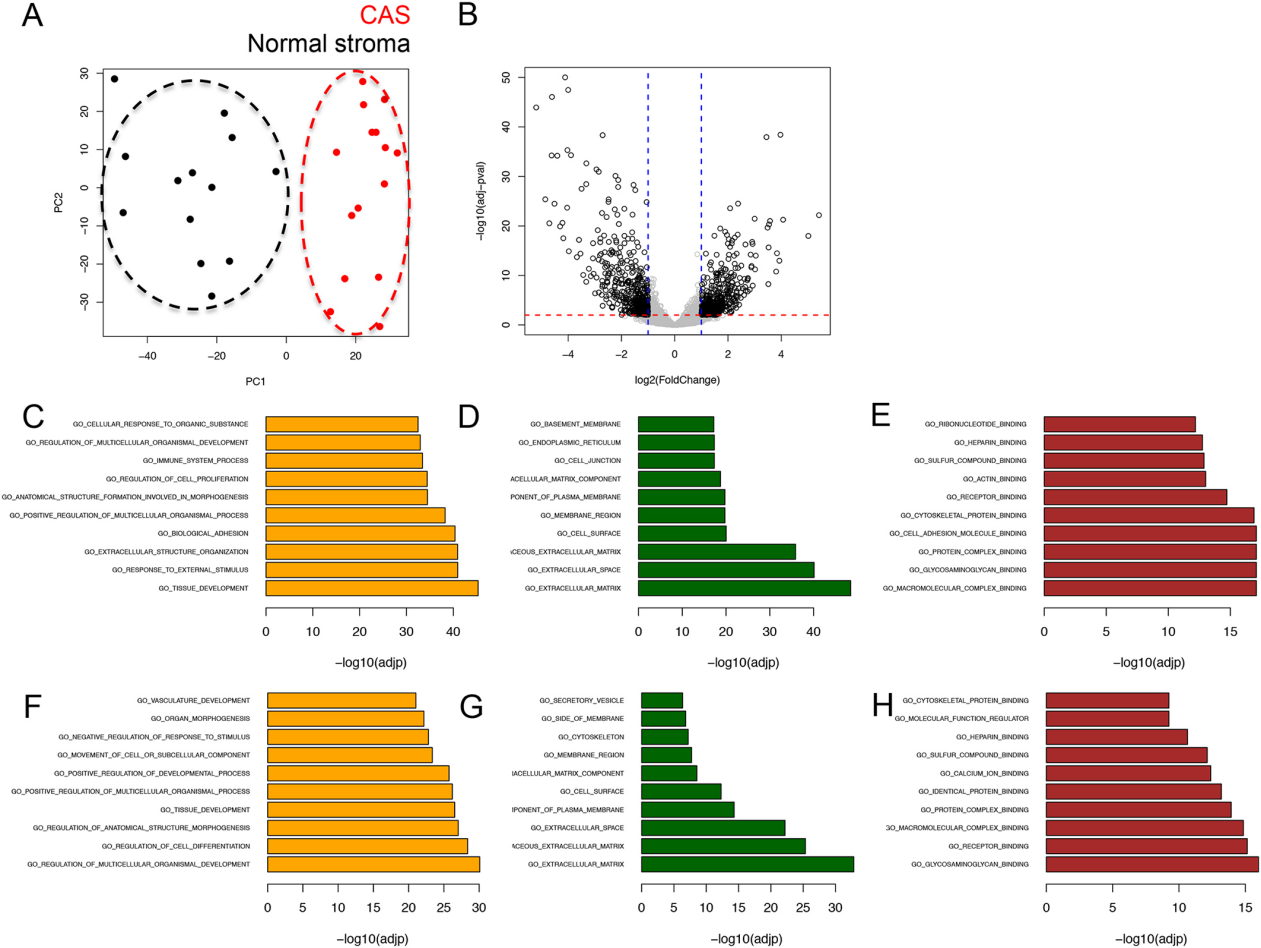
**RNAseq-based transcriptomic analysis of CAS and matched normal stroma from 15 canine simple mCA.** (A) PCA of CAS and normal stroma samples isolated from canine simple mCA. PCA was performed using all genes. (B) Volcano plot highlighting differentially expressed genes in CAS compared to normal stroma, using fold change >2 and FDR <0.01 as cut-off values. (C-E) Top 10 over-represented GO terms associated with biological processes (C), cellular components (D) and molecular functions (E) among genes significantly upregulated in CAS compared to normal stroma. (F-H) Top 10 over-represented GO terms associated with biological processes (F), cellular components (G) and molecular functions (H) among genes significantly downregulated in CAS compared to normal stroma.

**Fig. 2.**
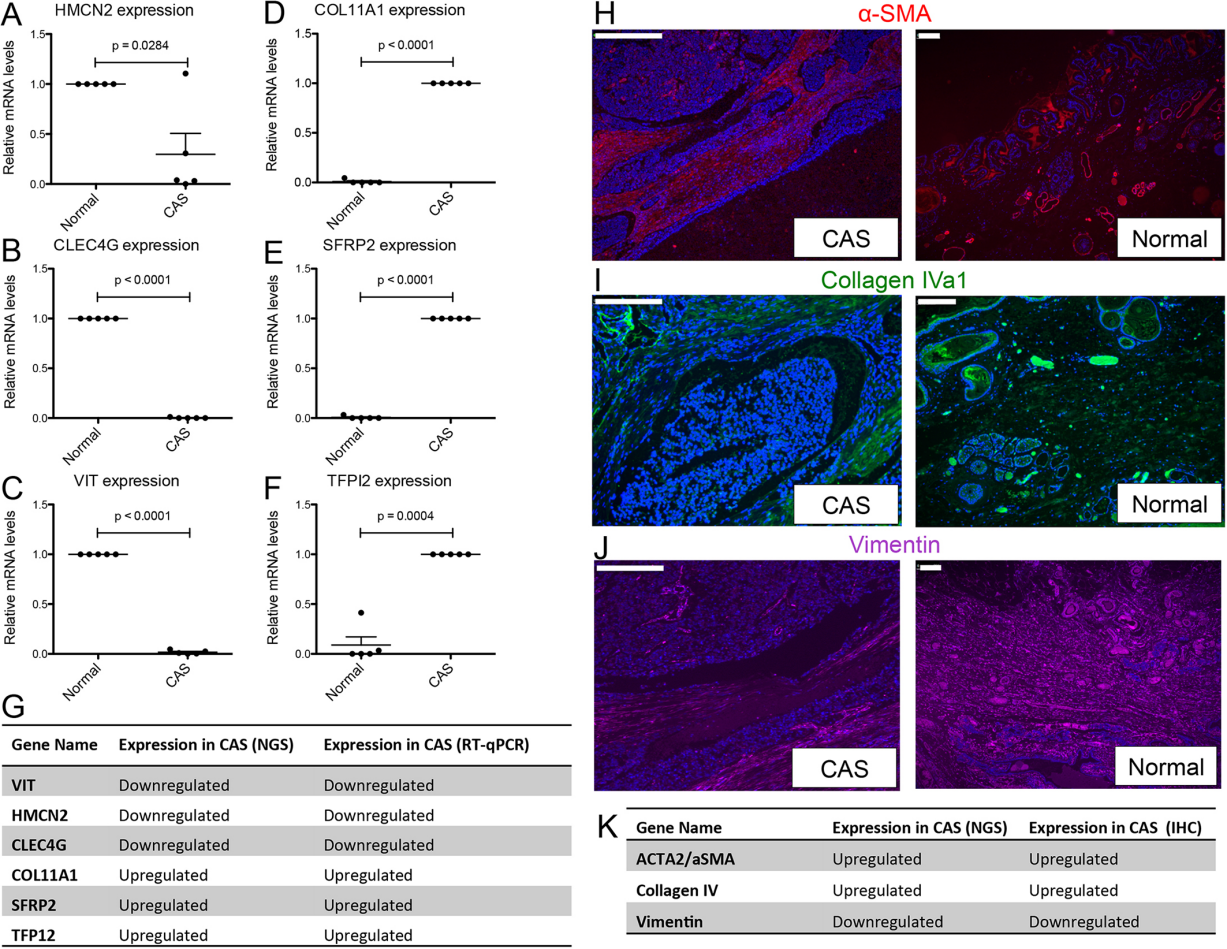
**Immunohistochemistry** **and RT-qPCR validation of selected genes from the carcinoma dataset.** (A-F) Relative mRNA levels of CAS-associated genes in normal stroma and CAS isolated by LCM, measured using RT-qPCR: *HMCN2* (A); *CLEC4G* (B); *VIT* (C); *COL11A1* (D); *SFRP2* (E); *TFPI2* (F). Data are mean±s.e.m., normalised to expression levels in normal stroma (for *HMCN2*, *CLEC4G* and *VIT*), or CAS (*COL11A1*, *SFRP2*, *TFPI2*), respectively. *P*-values were calculated using student's *t*-test, and significance cutoff was set at *P*=0.05. (G) Summary of the expression trends as detected by RT-qPCR and RNAseq. (H-J) Immunofluorescent staining of α-SMA (red, H), collagen IV (green, I) and vimentin (purple, J) in CAS and normal stroma of a representative canine simple mCA sample. DAPI staining (blue) visualises cell nuclei, and marks large densely blue areas in the left panels as tumour cells, while demarcating normal mammary glands in the panels on the right. (K) Summary of the expression trends as detected by RNAseq or immunofluorescence. Scale bars: 200 μm.

To analyse CAS from canine simple mCA, we concurrently isolated RNA from CAS and matched ‘normal’ stroma (i.e. stroma adjacent to unaltered mammary glands) from clinical FFPE specimens using LCM, and subjected these samples to RNAseq using our recently established protocol (Amini et al., 2017). Representative images for tissue isolation and patient characteristics for all cases included in the study can be found in Table 1 and Fig. S1. Remarkably, principal component analysis (PCA) revealed clear separation between CAS and normal stroma, highlighting the distinction between CAS and normal stroma as the major source of variability in the data (Fig. 1A). Differential expression analysis with a false discovery rate (FDR) cut-off of 0.01 and fold change threshold of 2 revealed 884 genes to be significantly deregulated in CAS compared to normal stroma, with 446 genes significantly upregulated and 438 genes significantly downregulated in CAS (Fig. 1B and Table S1). Over-representation analysis of Gene Ontology (GO) terms associated with biological processes, cellular components and molecular functions for up- and downregulated genes suggested the strongest changes in the following categories: immune system process, biological adhesion, cell differentiation, proliferation, growth, extra cellular matrix and collagen organisation (Fig. 1C-H). Association of significantly deregulated genes with the top over-represented GO terms revealed downregulated genes to participate in angiogenesis and upregulated genes to play a role in ‘cellular response to organic substance’, which is related to any process that results in a change in state or activity of a cell (in terms of movement, secretion, enzyme production, gene expression, etc.) as a result of an organic substance stimulus (Fig. S2). To validate the RNAseq data, we measured gene expression of six significantly differentially expressed genes (*HMCN2*, *CLEC4G*, *VIT*, *COL11A1*, *SFRP2* and *TFPI2*) by RT-qPCR. All of the tested genes showed significant expression changes consistent with the RNA-seq data (Fig. 2A-G). In addition, we validated differential expression in CAS versus normal stroma of three genes [upregulation of α-smooth muscle actin (α-SMA, encoded by *ACTA2*), upregulation of collagen 4α1 (*COL4A1*) and downregulation of vimentin in CAS compared to normal stroma] on protein level using immunofluorescence (Fig. 2H-K).

**Table 1.**
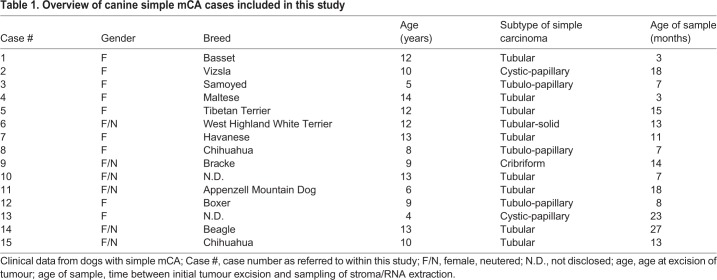
**Overview of canine simple mCA cases included in this study**

To enumerate the cellular landscape of tumour and normal stroma in canine mCA, we used xCell, a state-of-the-art algorithm that performs cell-type enrichment analysis from gene expression data for 64 immune and stroma cell types (Aran et al., 2017). As most deconvolution algorithms (including xCell) rely on human (and seldom mouse) gene signatures, we used the human orthologues of canine genes to perform *in silico* deconvolution. Fig. S3 shows differentially abundant cell types in CAS and normal stroma based on xCell enrichment scores. Unsupervised clustering of samples based on xCell enrichment scores revealed a clear separation between CAS and normal stroma, further corroborating our finding at the gene expression level. Taken together, these findings support the validity of our approach to analyse FFPE tissue subsections through LCM coupled with RNAseq, and demonstrate strong reprogramming to occur in CAS from canine mCA.

### CAS from canine and human mCA display a high grade of molecular homology

**Fig. 3.**
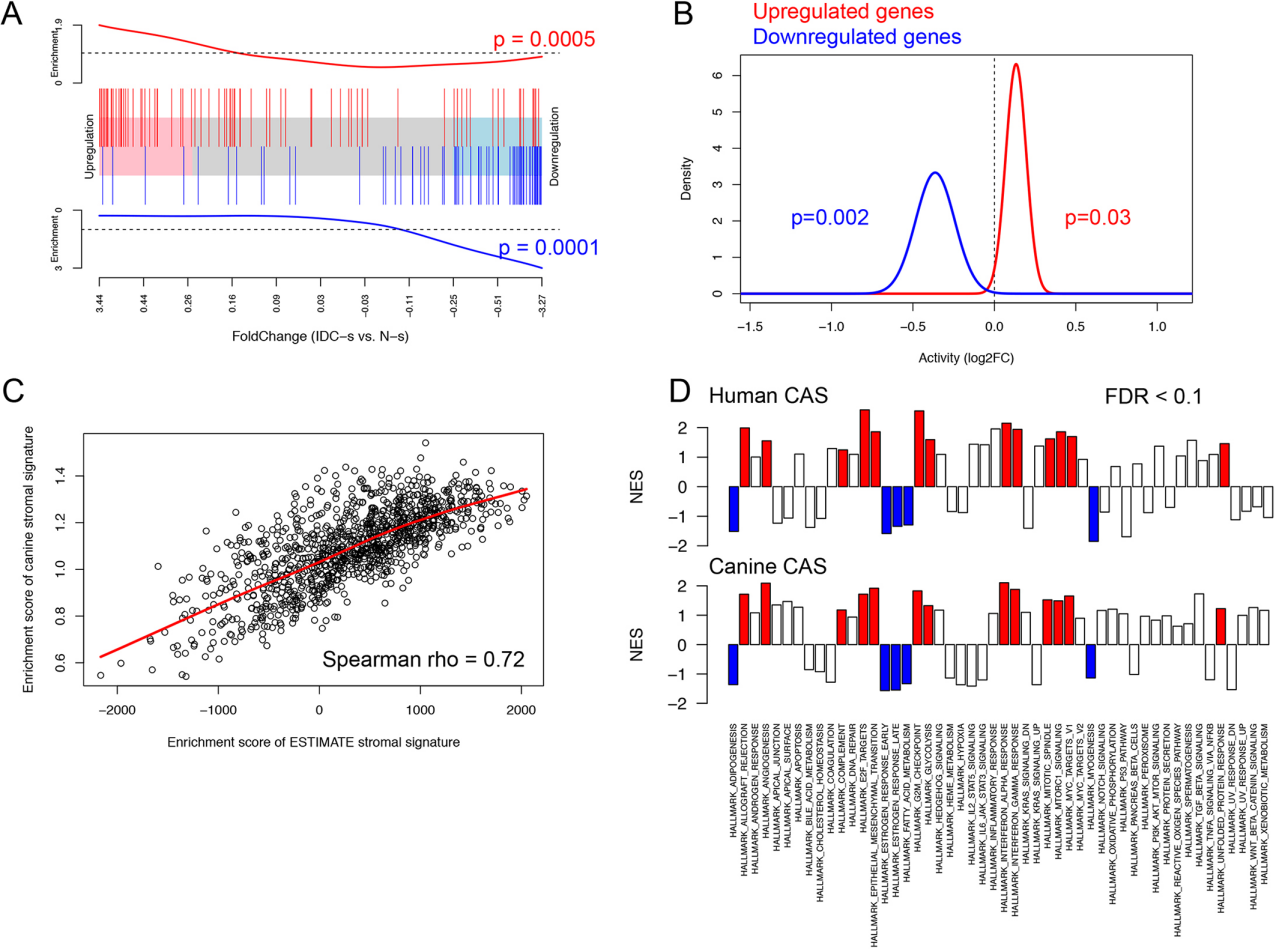
**CAS from canine and human mCA display a high grade of molecular homology.** (A) Competitive gene set testing to compare canine CAS to human CAS. GSEA-like running sum statistic depicting the location of selected genes on a ranked list of genes in human IDC stroma compared to normal stroma (GSE35019). Top 100 upregulated genes identified in canine tumour stroma are indicated as red vertical bars, top 100 downregulated genes identified in canine tumour stroma are indicated as blue vertical bars. (B) Self-contained gene set testing (QuSAGE method) to assess the average differential expression of canine-derived gene sets (i.e. up- and downregulated genes in canine CAS: 449 up- and 425 downregulated genes) in human breast cancer stroma. The *x*-axis demonstrates mean fold change expression in CAS compared to normal stroma in human mCA (GSE35019). The *y*-axis indicates the distribution of fold change expression within each set. *P*-values were calculated by comparing mean fold change to fold change of 1 using Welch's *t*-test. (C) Positive correlation (Spearman's rho=0.72) between the enrichment of significantly upregulated genes derived from canine data and the enrichment of an independently derived human stromal signature (ESTIMATE stromal signature; Yoshihara et al., 2013) in the breast cancer dataset from TCGA including >1000 cases. (D) GSEA analysis of hallmark pathways (MSigDB) in human data (GSE35019, top row) and canine data (bottom row). Red indicates pathways that were deemed significant (FDR<0.1) in both datasets and showed enrichment in CAS compared to normal stroma. Blue indicates pathways that were deemed significant (FDR<0.1) in both datasets and showed enrichment in normal stroma compared to CAS.

To gain insight into whether and to what degree CAS of canine and human mCA are comparable, we compared our dataset with a similar published human breast cancer patient dataset (GSE35019) consisting of matched stromal samples of invasive ductal carcinoma (IDC), ductal carcinoma *in situ* (DCIS) and normal stroma (Vargas et al., 2012). We postulated that if canine and human mCA were to share a high level of molecular homology, differentially regulated genes in canine CAS versus normal stroma should exhibit a similar expression pattern in the human dataset. To test this hypothesis, we ranked all genes in the human dataset based on fold change expression in IDC stroma versus normal stroma. We then assessed the enrichment of canine-derived CAS signature in this ranked gene list. Remarkably, we found the upregulated subset of the canine CAS signature to be enriched on the left-hand side of the ranked gene list (*P*-value <0.001), demonstrating overexpression in IDC stroma (Fig. 3A, showing the top 100 up- and downregulated genes from the canine CAS signature in the ranked human gene list, and Fig. S4 for all genes). In contrast, the downregulated subset of the canine CAS signature was enriched on the right-hand side (*P*-value <0.001), which is associated with normal stroma. Similar results were obtained using an alternative gene set testing procedure (Fig. 3B). As a complementary approach to interrogate the molecular homology between canine and human mCA, we used a well-established and manually curated set of stromal genes obtained using human samples (Yoshihara et al., 2013) and computed the stromal enrichment scores using single sample Gene Set Enrichment Analysis (ssGSEA) (Vargas et al., 2012), an extension of the popular GSEA method that calculates the extent at which members of a given gene set are co-ordinately up- or downregulated within individual samples. Using this approach, we found the enrichment of the canine-derived stromal signature to be highly positively correlated (Spearman rho=0.72, *P*-value <0.001) with the enrichment of the human-derived stromal signature in the breast cancer subset of TCGA (sample size >1000), further supporting the presence of strong molecular homology in CAS between the two species (Fig. 3C). To further explore the molecular similarities between canine and human mCA, we sought to identify perturbations in signalling pathways present in CAS of both species. Enrichment analysis of hallmark pathways showed a substantial number of pathways to be significantly deregulated in the same direction in both human and canine CAS (Fig. 3D). The commonly perturbed pathways include angiogenesis, epithelial mesenchymal translation, glycolysis, allograft rejection, interferon gamma and alpha response, complement, estrogen response late and early, adipogenesis, fatty acid metabolism, E2F targets, G2M checkpoint, MYC targets, mitotic spindle, unfold protein response, myogenesis and mTORc pathway. GSEA leading edge subsets of genes deriving the conserved perturbations observed in both species are detailed in Table 2. To sum up, these results clearly demonstrate the presence of significant molecular homology between CAS in canine mCA and CAS from human breast cancers.

**Table 2.**
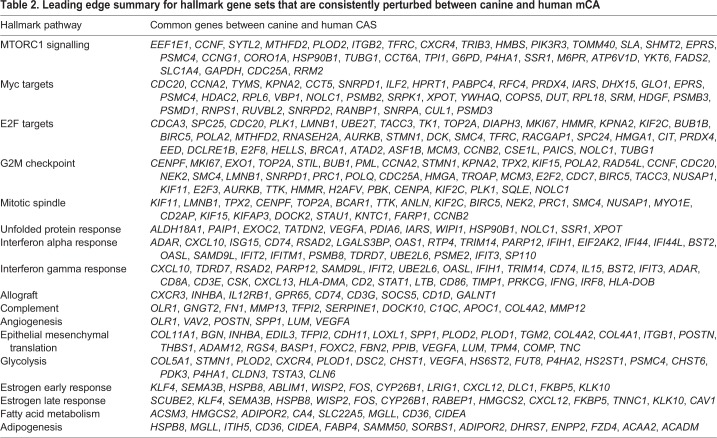
**Leading edge summary for hallmark gene sets that are consistently perturbed between canine and human mCA**

### The human orthologues of the canine-derived CAS signature are enriched among highly prognostic genes in human mCA

**Fig. 4.**
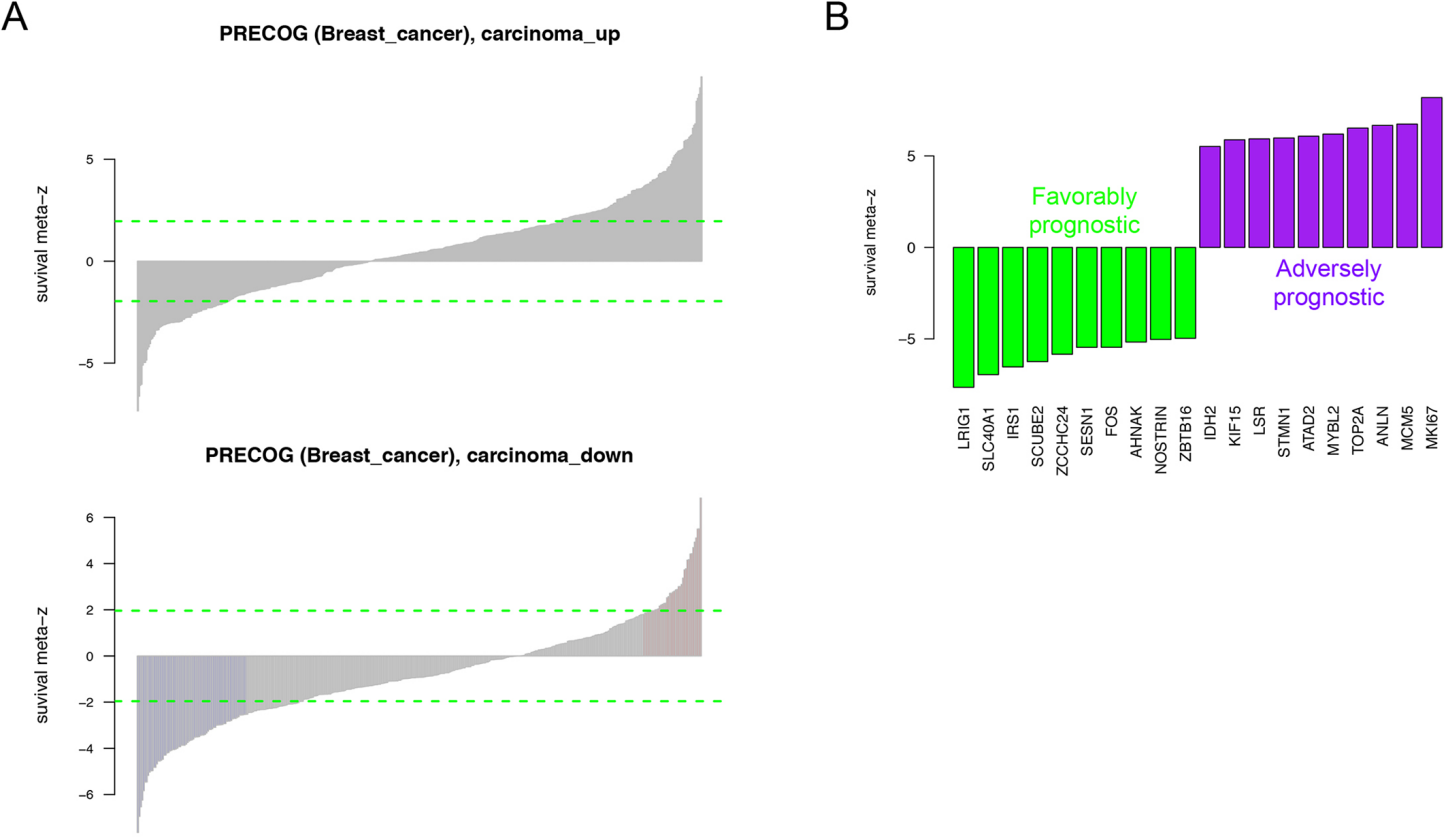
**Human orthologues of genes derived from the canine CAS signature are enriched among highly prognostic genes in human mCA.** (A) Breast cancer meta *z*-scores of the human orthologues of canine-derived CAS genes obtained from PRECOG database (Gentles et al., 2015).Top: genes upregulated in CAS are enriched for positive meta *z*-scores, associated with adverse prognosis. Bottom: genes upregulated in normal stroma are enriched for negative meta *z*-scores, associated with favourable prognosis. Refer to Table S2 for the full list of meta *z*-scores. (B) Stromal signature of canine mCA shows strong association with survival in human breast carcinomas. Survival meta *z*-scores were obtained from PRECOG database (Gentles et al., 2015). A positive *z*-score indicates an adversely prognostic gene, whereas a negative *z*-score indicates a favourably prognostic gene. Data shown for 20 genes with strongest association with survival. Refer to Table S2 for the full list of meta *z*-scores.

The tumour stroma has important roles in development, progression and metastasis of different tumour types, and targeting both cancer cells and the stromal compartment is thought to increase efficiency of anticancer therapy and improve patient survival (Valkenburg et al., 2018). Given the scarcity of long-term survival data from the canine cases included in this analysis, we set out to explore the clinical relevance of our canine-derived CAS signature by exploring the association with survival of their human orthologues using the PRECOG database (Gentles et al., 2015). PRECOG enables querying the association between expression of a given gene and patient survival using aggregate data from multiple studies via survival meta *z*-scores. We hypothesised that high levels of molecular homology between canine and human mCA should manifest in a prognostic value of the canine CAS signature. Reassuringly, we found that upregulated genes in canine CAS were highly enriched among adversely prognostic genes (pre-ranked GSEA *P*-value <0.001), whereas upregulated genes in normal stroma were highly enriched among favourably prognostic genes (pre-ranked GSEA *P*-value <0.001). Fig. 4A shows the full list of genes, and Fig. 4B displays the top 20 genes from the canine CAS signature, with the largest meta *z*-scores indicating the strongest association with survival (for the full list of genes see Table S2). Note that the positive and negative meta *z*-scores as described in Gentles et al. (2015) indicate adversely and favourably prognostic genes, respectively. In conclusion, our findings suggest that the molecular homology between CAS from human and canine mCA is strongly rooted in conservation of key signalling pathways that underlie the prognostic value of CAS-derived gene expression changes in both species, thus further emphasising the merit of canine mCA as a model for human mCA.

## DISCUSSION

Archival FFPE tissue samples constitute a vast and very valuable collection of patient samples for biomedical research. However, owing to chemical cross-linking of macromolecules, extraction of RNA from these tissues is challenging in terms of quantitative and qualitative constraints, which heavily impact on downstream analysis. Despite these drawbacks, several studies have proven that it is feasible to analyse RNA or miRNA from FFPE with RNAseq, and that results from FFPE are largely consistent with fresh-frozen material (e.g. Hedegaard et al., 2014; Meng et al., 2013; Sinicropi et al., 2012). However, all of these studies relied on rather large amounts of input tissue (3-6 full-size sections of 10 μm thickness, or 5-10 mg tissue) and between 100-500 ng of RNA. When, as an additional constraint, sample size is limiting, such as when performing laser-capture microdissection (LCM) to isolate specific subpopulations of cells, recovery of sufficient RNA for RNAseq-based analysis pipelines becomes virtually impossible, effectively precluding RNAseq-based approaches of small FFPE samples. To enable utilisation of valuable archival FFPE tissue samples and to be able to analyse CAS reprogramming in canine mCA, we have recently established a protocol for RNA extraction from LCM-FFPE tissue to be amenable to analysis by RNA-seq (Amini et al., 2017). Using this novel protocol, which routinely uses only 4 ng of input RNA, we have now analysed CAS and matched normal stroma isolated by LCM from FFPE tissue from 15 clinical cases of canine simple mCA. Importantly, this approach is not limited to isolation of CAS, but can be leveraged to analyse almost any subsection of interest from FFPE tissues. Naturally, the protocol can also be applied to entire sections of FFPE material that have not undergone microdissection. Historically, LCM-based interrogation of human CAS and other tissue subsections has been performed mainly through analysis of fresh-frozen tissue sections (Erickson et al., 2009). Although feasible, procurement of fresh-frozen tissue sections of a given pathology necessitates a high grade of coordination between treating physicians and researchers, and can usually only be performed in prospective settings, as fresh freezing of tissue is not normally integrated into the diagnostic standard workflow. Also, storage and handling of fresh-frozen tissue is more cost- and labour-intensive than that of standard FFPE blocks. In addition, standard diagnostic routine workflows strongly depend on FFPE tissue, because of good tissue morphology and ease of handling, which results in production of large amounts of FFPE tissue specimens in pathology departments that can be stored almost indefinitely at room temperature.

CAS plays a key role in cancer initiation and progression in human cancer (Bissell and Hines, 2011; Hanahan and Coussens, 2012), and thus it likely occupies a central role in the biology of canine tumours as well. Spontaneous canine mCA are often regarded as valuable models for human mCA, but the lack of data on canine CAS and thus unbiased cross-species analysis of molecular homologies and differences undermine the validity of these assumptions. To date, however, CAS in canine cancers in general, and canine mCA in particular, entirely lacks systematic characterisation. We have recently analysed the expression of a small panel of genes known to be deregulated in human CAS by qRT-PCR in CAS from canine simple mCA (Ettlin et al., 2017). These initial results suggested strong overlaps in CAS-related biology between canine and human mCA. Here, we provide the first dataset for CAS and matched normal stroma in canine mCA obtained through systematic RNAseq-driven transcriptomic characterisation (Figs 1,2). Our results reveal strong reprogramming in CAS from canine mCA. These results will serve as a basis for further mechanistic follow-up studies of the involvement of stromal genes in development and progression of canine mCA, and have the potential to reveal novel prognostic markers and therapeutic targets.

Our unbiased approach has yielded highly interesting insights into the degree of cross-species homology of CAS reprogramming between human and canine mCA. We demonstrate that reprogramming of CAS from human and canine mCA is highly comparable, and relationships between different gene signatures observed in human breast cancer are largely maintained in the canine model, suggesting a close interspecies similarity in the network of cancer signalling circuitries (Fig. 3). As an example, in the context of angiogenesis, we found that vascular endothelial growth factor A (*VEGFA*), secreted phosphoprotein 1/osteopontin (*SPP1*), periostin (osteoblast specific factor; *POSTN*) and VAV guanine nucleotide exchange factor (*VAV2*) are important commonly regulated genes between human and canine CAS. All of these are known to enhance tumour progression in cancer in general, and mCA in particular (Hunter et al., 2006; Morishita, 2011; Queiroga et al., 2011a; Wang and Ouyang, 2012). Various studies investigating spontaneous mCA of both canine and human origin support the role of the epithelial-to-mesenchymal transition (EMT) in metastasis in both species (Lamouille et al., 2014; Raposo-Ferreira et al., 2018; Thiery, 2002). Indeed, cells in CAS have been shown to be potent inducers of EMT (Jing et al., 2011). Furthermore, the collagen-associated genes *COL11A1*, *COL4A1/2* and *ADAM12*, which we identified as commonly regulated between both species, are known to play an important role in EMT. *COL11A1* mRNA is significantly increased in various types of cancers (Raglow and Thomas, 2015), and its overexpression in CAS is associated with poor prognosis in human mCA (Parl et al., 1984). Expression of type IV collagen is related to tumour malignancy and fibroblast infiltration (Chen et al., 2015). Indeed, the levels of type IV collagen protein were strongly increased in CAS from canine mCA (Fig. 2H,K). Among the common glycolytic genes, *PLOD1/2* and *FUT8* are among the most important deregulated targets. Activation of *PLOD1* and *PLOD2* at the transcription level by HIF-1 is required for biogenesis of collagen in breast cancer cells (Gilkes et al., 2013). By remodelling the TGF-β receptor core fucosylation, FUT8 expression influences breast cancer invasiveness (Tu et al., 2017). Immune-related pathways significantly deregulated in both species include interferon alpha and gamma responses, allograft rejection and complement. Here, CXCL10, its receptor CXCR3, and CD74 were detected in most immune-related pathways in both human and canine CAS. The chemokine interferon gamma inducible protein CXCL10 is a member of the CXCR3 chemokine family that interconnects with various signalling pathways. In breast cancer, Ras-induced CXCL10 overexpression contributes to the development of breast tumours (Parl et al., 1984), and overexpression of CXCR3 is associated with poorer overall survival (Bronger et al., 2017). Expression of CD74, a chaperone protein with an important role in innate immunity, in breast cancer tissue is correlated with increased metastasis (Metodieva et al., 2013; Nieto et al., 2000). We also found hormone-related pathways, such as estrogen response, enriched in both human and canine CAS. In humans, benign tumours are likely to be estrogen receptor (ER)-positive with a better prognosis (Maynard et al., 1978), whereas ER-negative tumours are more likely to be of higher histological grade (Parl et al., 1984; Putti et al., 2004). The effect of ER expression on prognosis of canine mCA remains unclear (De las Mulas et al., 2005; Nieto et al., 2000). Likewise, the hormonal classification as a predictive marker of favourable response to endocrine therapy has not been established in canine mammary tumours, therefore, expression of hormone receptors analysis is currently not routinely assessed in canine mCA. Other common pathways include fatty acid metabolism, MTORC1 signalling, MYC targets v1, E2F targets, G2M checkpoint, mitotic spindle and unfolded protein response (Corazzari et al., 2017; Jardé et al., 2008; Lan et al., 2018; Pease and Tirnauer, 2011; Poole and van Riggelen, 2017; Silvera et al., 2017; Wang et al., 2009).

Finally, we establish the prognostic potential of the canine CAS signature in human samples (Fig. 4). These analyses strongly support the notion that CAS from canine and human mCA are highly comparable on a molecular level, suggesting similar reprogramming mechanisms underlying the development of CAS in human and canine mCA.

In conclusion, we provide a proof-of-principle to analyse specific subsections of FFPE tissue isolated through LCM by RNAseq, which unlocks a new dimension of difficult-to-analyse samples that now become amenable to investigation. This first study to systematically analyse gene expression in CAS and normal stroma from canine mCA reveals detailed insights into the biology of CAS in both canine tumour types and their comparability with the human counterpart, which support the validity of spontaneous canine mCA as a model to identify disease-promoting features with relevance to the human disease.

## MATERIALS AND METHODS

### Ethics approval and consent to participate

No animals were killed for the purpose of this research project, as the tissue analysed had been surgically removed in a curative setting with the verbal consent of the patient owners. According to the Swiss Animal Welfare Law Art. 3c, Abs. 4 the preparation of tissues in the context of agricultural production, diagnostic or curative operations on the animal or for determining the health status of animal populations is not considered an animal experiment and, thus, does not require an animal experimentation license. All the FFPE specimens used were obtained for diagnostic reasons and do therefore not require a formal ethics approval, in full compliance with national guidelines.

### Cases selection and tissue processing

Fifteen dog mammary carcinoma samples were obtained from the Institute of Veterinary Pathology of the Vetsuisse Faculty Zürich (Table 1). All samples were archival FFPE tissue samples either from the Small Animal Hospital of Zurich or external cases sent in by veterinarians practicing in Switzerland. Details regarding selection criteria are described in Ettlin et al. (2017). Paraffin blocks were routinely kept at room temperature. Tissue processing for LCM was performed as described in Amini et al. (2017). All cases were reviewed by a veterinary pathologist. The criteria for carcinoma case selection were: female dogs, simple type of mCA, appropriate tumour stroma content and areas with no obvious or only negligible inflammation. Table 1 provides clinical details, such as age and breed of each patient, sample age and tumour subtype, for all cases included in the study.

### Laser-capture microdissection

LCM was used for selective isolation of matched CAS and normal stroma from 15 canine simple mCA. LCM was performed using the ArcturusXT™ Laser Capture Microdissection System (Thermo Fisher Scientific) and Arcturus^®^ CapSure^®^ Macro LCM Caps (Life Technologies) as described previously (Amini et al., 2017; Ettlin et al., 2017). Areas for dissection were reviewed by a veterinary pathologist. Highly enriched populations of normal or tumour-associated stroma from the specimens were identified and isolated according to the manufacturer's protocol. Normal stroma samples were isolated from the same slides, from regions specified by a pathologist that presented no obvious alterations and were at least 2-4 mm away from the tumour, in accordance with established procedures (Finak et al., 2008). Isolation of cells of interest was verified by microscopic examination of the LCM cap as well as the excised region after microdissection (Fig. S1).

### RNA isolation

RNA was isolated using the Covaris^®^ truXTRAC FFPE RNA kit and the Covaris^®^ E220 focused ultrasonicator as described in Amini et al. (2017). An overview of concentration, total yield and DV200 for all samples can be found in Table S5.

### Quantitative RT-PCR

Quantitative RT-PCR using Taqman^®^ primers was performed as described in Amini et al. (2017). Primers are detailed in Table S3.

### Immunofluorescence

FFPE tissue sections (2 µm thickness) were mounted on positively charged slides and dried overnight at 37°C. Drying was followed by the deparaffinisation of the slides with four xylene baths for 5 min each using the Tissue-Tek^®^ Prisma^®^ and Film^®^ (Sysmex). For rehydration, a degressive alcohol series using 100% ethanol, 95% ethanol, 70% ethanol and distilled water was performed.

All immunofluorescence sections were counterstained with 0.1 mg/ml DAPI (Thermo Fisher Scientific) at room temperature for 15 min to visualise nuclei. Antibodies and conditions used for immunofluorescence are detailed in Table S4.

### RNA-seq library preparation

RNAseq was carried out on 4 ng of RNA from elution 1 (E1) diluted to a concentration of 0.33 ng/μl in a total volume of 30 μl. The SMARTer Stranded Total RNAseq Kit-Pico Input Mammalian (Clontech/Takara Bio USA) was used according to manufacturer's protocol for RNA library preparation and ribosomal RNA depletion. Single-read sequencing (125 bp) was obtained using the Illumina HiSeq 4000 according to standard protocols of the Functional Genomics Centre Zurich. See Fig. S5 for a barplot of library sizes demonstrating the number of quantified reads per sample.

### Bioinformatics analyses

RNA-seq quantification was performed using kallisto 0.44.0 with sequence-based bias correction using transcript sequences obtained from ENSEMBLE (CanFam3.1) (Bray et al., 2016). All other parameters were set to default when running kallisto. Kallisto's transcript-level estimates were further summarised at the gene-level using tximport 1.8.0 from Bioconductor (Soneson et al., 2015). Both raw data and gene-by-sample matrix of estimated counts were deposited online. For downstream analysis, lowly abundant genes were filtered out and unwanted variation was estimated using SVA 3.30.1 from Bioconductor (Leek et al., 2019). Differential expression analysis was performed using DESeq2 1.22.0 from Bioconductor (Love et al., 2014), with estimated factors of unwanted variation included as additional covariates in the design formula. Significant genes were identified using FDR and fold-change thresholds as indicated per dataset. Heatmaps were generated using the pheatmap R Package (Kolde, 2019), with clustering distance and method set to Euclidean and ward.D2, respectively. Over-representation analysis of differentially expressed genes was performed using the MSigDB webtool (http://software.broadinstitute.org/gsea). GSEA was performed using fgsea 1.8.0 from Bioconductor (Sergushichev, 2016), with signal-to-noise ratio as defined by Subramanian et al. (2005) as gene-level statistic. Before performing GSEA, dog genes were converted to human orthologues using biomaRt 2.38.0 from Bioconductor (Durinck et al., 2009). If a human orthologue was associated with more than one dog gene, the dog gene with maximum variance was selected using the collapseRows functionality within the WGCNA R package (Miller et al., 2011). Signalling pathways analysed by GSEA were obtained from the Hallmark gene sets of the MSigDB (Liberzon et al., 2015). Processed microarray data was obtained from Gene Expression Omnibus under accession number GSE35019. The barcode enrichment plot was generated using the barcodeplot functionality, and significance was assessed using the geneSetTest functionality, both within limma 3.38.3 from Bioconductor (Ritchie et al., 2015). The human stromal gene signature was obtained from the ESTIMATE R package (Yoshihara et al., 2013). TCGA processed data was obtained from cbioportal data portal (Gao et al., 2013), and the enrichment of canine and human stromal signatures in TCGA's BRCA data was computed using ssgsea functionality within GSVA 1.30.0 from Bioconductor (Hänzelmann et al., 2013). Survival meta *z*-scores were obtained from PRECOG database (https://precog.stanford.edu, Gentles et al., 2015). Enrichment of canine CAS signature among highly prognostic genes was assessed using pre-ranked GSEA as implemented in fgsea 1.8.0 from Bioconductor (Sergushichev, 2016), with PRECOG's Breast Cancer meta *z*-scores as gene-level statistic.

## Supplementary Material

Supplementary information
