# Supplementary material for: Next-generation RNA sequencing of FFPE subsections reveals highly conserved stromal reprogramming between canine and human mammary carcinoma
Source: Dis Model Mech. 2019 Aug 8;12(8):dmm040444. doi: 10.1242/dmm.040444 (PMC6737962; doi:10.1242/dmm.040444)
Supplement: Supplementary information [file dmm-12-040444-s1.pdf]

Figure S1: Selective isolation of cancer-associated stroma and normal stroma from canine simple mammary carcinomas by laser-capture-microdissection. Representative images of tissue mounted on the slide to validate proper isolation of stromal cells were taken at x 10 magnification before dissection (A, D), after dissection (B, E), and of the cap containing the excised tissue sections (C, F). Images are from one representative case of simple mammary carcinoma: CAS (A-C) and normal stroma (D-F).

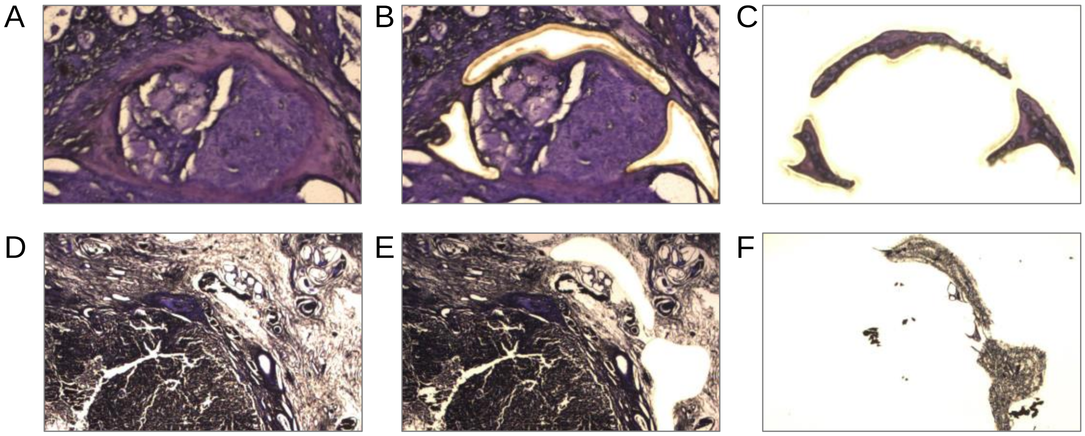

Disease Models & Mechanisms • Supplementary information

Figure S2: Over-representation analysis of GO terms among significant genes in carcinoma data. Association of significantly de-regulated genes identified in CAS compared to normal stroma in canine mammary carcinoma, with top 10 over-represented Gene Ontology (GO) terms associated with biological processes, cellular components, and molecular functions as presented in Figure 1D-I.

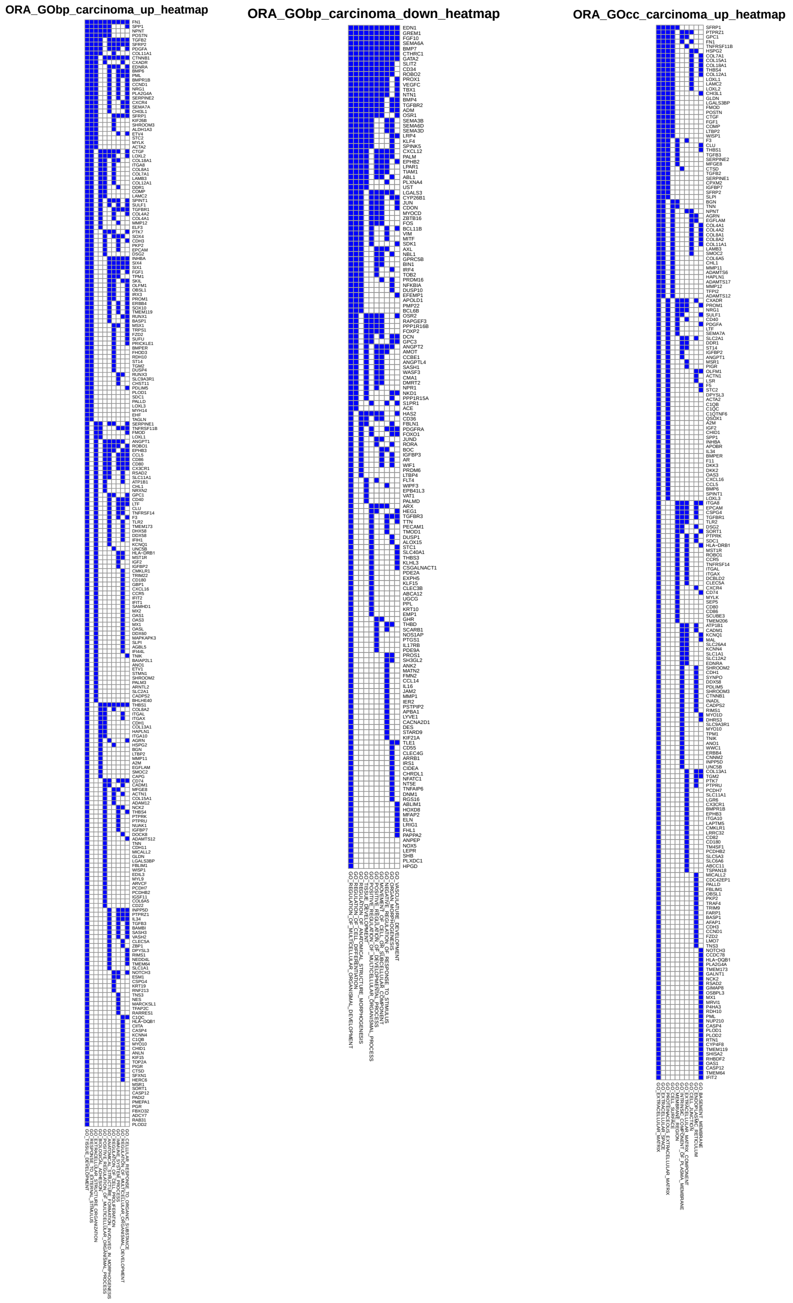

ORA_GOcc_carcinoma_down_heatmap

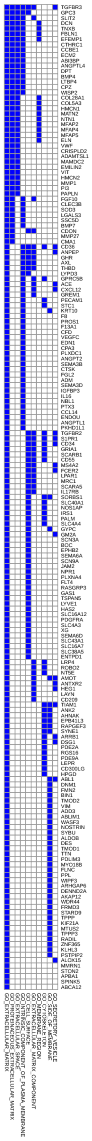

ORA_GOmf_carcinoma_up_heatmap

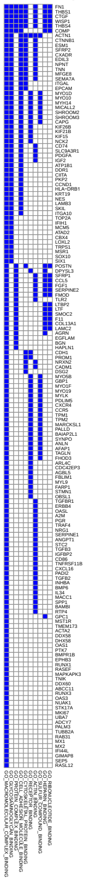

ORA_GOmf_carcinoma_down_heatmap

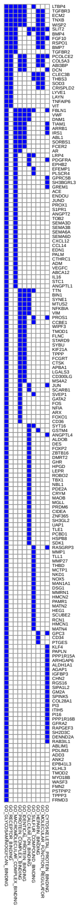

Figure S3: Deconvolution of the cellular landscape of CAS and normal stroma in canine mammary carcinomas by xCell. above) Deconvolution of the cellular landscape in CAS and normal stroma samples. **below)** List of abbreviations used by xCell for the different cell types analysed.

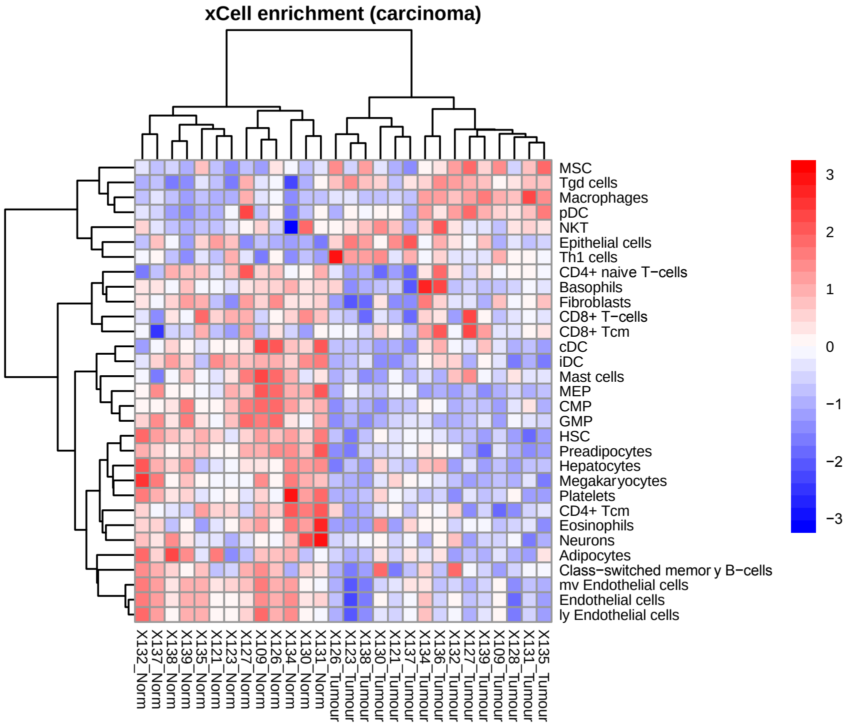

Full name	Reported
Monocytes	Monocytes
CD8+ T-cells	CD8+ T-cells
NK cells	NK cells
Macrophages	Macrophages
Endothelial cells	Endothelial cells
Dendritic cells	DC
Neutrophils	Neutrophils
Erythrocytes	Erythrocytes
CD4+ naive T-cells	CD4+ naive T-cells
Multipotent progenitors	MPP
Smooth muscle cells	Smooth muscle cells
Fibroblasts	Fibroblasts
Epithelial cells	Epithelial cells
Keratinocytes	Keratinocytes
Chondrocytes	Chondrocytes
Adipocytes	Adipocytes
B-cells	B-cells
CD4+ T-cells	CD4+ T-cells
CD8+ effector memory T-cells	CD8+ Tem
Common myeloid progenitors	CMP
Granulocyte-macrophage progenitors	GMP
Megakaryocyte–erythroid progenitors	MEP
Regulatory T-cells	Tregs
Hematopoietic stem cells	HSC
Plasma cells	Plasma cells

CD4+ central memory T-cells	CD4+ Tcm
Microvascular endothelial cells	mv Endothelial cells
CD4+ effector memory T-cells	CD4+ Tem
Memory B-cells	Memory B-cells
CD8+ central memory T-cells	CD8+ Tcm
naive B-cells	naive B-cells
Eosinophils	Eosinophils
Macrophages M1	Macrophages M1
Myocytes	Myocytes
Lymphatic endothelial cells	ly Endothelial cells
Mesenchymal stem cells	MSC
Macrophages M2	Macrophages M2
Osteoblasts	Osteoblast
Activated dendritic cells	aDC
Preadipocytes	Preadipocytes
Melanocytes	Melanocytes
Skeletal muscle cells	Skeletal muscle cells
CD4+ memory T-cells	CD4+ memory T-cells
Megakaryocytes	Megakaryocytes
pro B-cells	pro B-cells
Basophils	Basophils
Conventional dendritic cells	cDC
Astrocytes	Astrocytes
Plasmacytoid dendritic cells	pDC
Pericytes	Pericytes
Neurons	Neurons
Class-switched memory B-cells	Class-switched memory B-cells
Hepatocytes	Hepatocytes
Mesangial cells	Mesangial cells
Immature dendritic cells	iDC
Mast cells	Mast cells
Type 2 T-helper cells	Th2 cells
Common lymphoid progenitors	CLP
Platelets	Platelets
Type 1 T-helper cells	Th1 cells
CD8+ naive T-cells	CD8+ naive T-cells
Natural killer T-cells	NKT
Sebocytes	Sebocytes
Gamma delta T-cells	Tgd cells

Figure S4: Cancer-associated stroma from canine and human mammary carcinomas displays a high grade of molecular homology. Competitive gene set testing to compare canine CAS to human CAS. GSEA-like running sum statistic depicting the location of selected genes on a ranked list of genes in human IDC stroma compared to normal stroma (GSE35019). All upregulated genes identified in canine tumor stroma are indicated as red vertical bars, all down-regulated genes identified in canine tumor stroma are indicated as blue vertical bars.

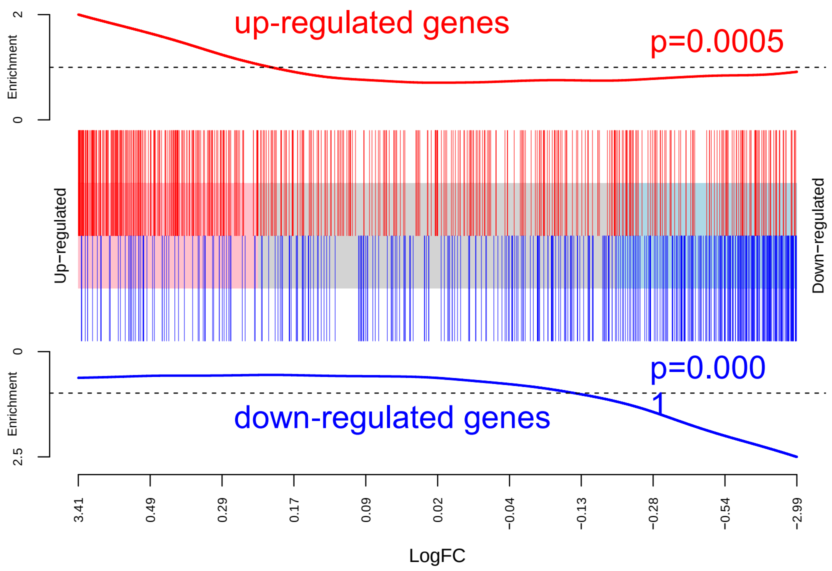

Figure S5: Barplot of library sizes demonstrating the number of quantified reads per sample.

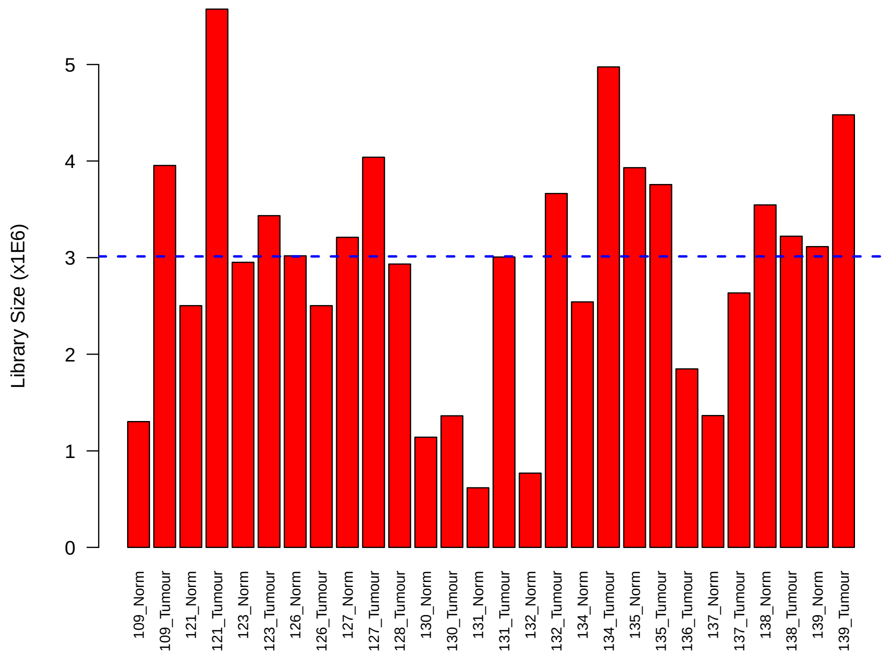

Table S1. Differentially expressed genes for CAS from canine carcinoma.

Click here to Download Table S1

Table S2. Full list of genes with breast cancer meta z-scores of the human orthologs of canine-derived CAS genes obtained from PRECOG database.

Click here to Download Table S2

Disease Models & Mechanisms • Supplementary information

Table S3. List of primers used for qRT-PCR. The "c" before each gene indicates that primers were designed to detect the canine isoforms of the intended targets.

Gene Target	Sequence	Amplicon Length (nt)	Taqman® Order Number or custom design reference
cGAPDH	Fw: 5'-GCTGCCAAATATGACGACATCA-3'<br>Rev: 5'-GTAGCCCAGGATGCCTTTGAG-3'<br>Probe: 5'-TCCCTCCGATGCCTGCTTCACTACCTT-3'	75	-
cPPIA	Manufacturer's proprietary information	92	Cf03986523_gH
cB2M	Manufacturer's proprietary information	87	Cf02659077_m1
cHMCN2	Manufacturer's proprietary information	76	Cf02654692_m1
cCOL11A1	Manufacturer's proprietary information	54	ARCE4CX
cCLEC4G	Manufacturer's proprietary information	69	Cf02647951_g1
cSFRP2	Manufacturer's proprietary information	110	Cf02625033_m1
cVIT	Manufacturer's proprietary information	68	Cf02657286_m1
cTFPI2	Manufacturer's proprietary information	88	Cf02642366_m1
cSTRA6	Manufacturer's proprietary information	61	Cf02661996_m1
cIGF2	Manufacturer's proprietary information	111	Cf02647135_m1
cPIGR	Manufacturer's proprietary information	70	Cf02625115_m1
cSFRP1	Manufacturer's proprietary information	69	Cf02654440_m1
cSCUBE2	Manufacturer's proprietary information	77	Cf02632274_m1
cMMP2	Manufacturer's proprietary information	58	Cf02623423_m1
cSDK1	Manufacturer's proprietary information	75	ARPRKXN

Table S4. Antibodies and conditions used for immunofluorescence. Basic/acidic antigen retrieval indicate 20 min incubation in pH 9.0 EDTA buffer or pH 6.0 citrate buffer in a pressure cooker set to 98°, respectively.

**Primary antibody**					**Secondary antibody conditions**
Antigen	Type	Source	Antigen retrieval	Dilution And incubation	
SMA (anti human)	Monoclonal Mouse	M0851, Agilent	None	1:400, RT, 1h	goat anti mouse (Invitrogen A11005, 594) 1:400, 1 hour, RT
Vimentin (anti mouse)	Monoclonal Mouse	M7020, Agilent	Basic	1: 150, RT, 1h	goat anti mouse (Invitrogen A11005, 594) 1:400, 1 hour, RT
Collagen IV (anti mouse)	Monoclonal Mouse	Bio-Rad 2150-1470	Basic	1:50,4°C , over night	goat anti rabbit (Invitrogen A11012, 594) 1:400, 1 hour, RT

Disease Models & Mechanisms • Supplementary information

Table S5. Summary of RNA yield and quality of samples used in this study.

Case #	Sample	Concentration (pg/ul)	Total yield (ng)	DV200 (%)
1	Normal	2060	62	79.68
	CAS	5870	176	81.54
2	Normal	4020	120	88.11
	CAS	3960	118	86.92
3	Normal	322	10	75.41
	CAS	2340	70.2	75.39
4	Normal	392	11.76	69.57
	CAS	1200	36	77.09
5	Normal	1210	36.3	68.02
	CAS	485	14.5	70.06
6	Normal	443	11.1	72.82
	CAS	1680	42	80.63
7	Normal	1480	44.4	83.55
	CAS	2870	86.1	82.85
8	Normal	1330	39.9	82.10
	CAS	1000	30	78.73
9	Normal	1170	35.1	71.67
	CAS	3440	103.2	71.85
10	Normal	1540	46.2	85.53
	CAS	1940	58.2	80.88
11	Normal	895	26.8	69.73
	CAS	1840	55.2	74.59
12	Normal	565	17	78.63
	CAS	1240	37.2	83.47
13	Normal	405	12.1	70.41
	CAS	1220	36.6	72.67
14	Normal	901	27	77.79
	CAS	6960	209	79.17
15	Normal	1340	40.2	77.80
	CAS	7000	210	75.22

Disease Models & Mechanisms • Supplementary information
